# Analyzing the Robustness of Complex Networks with Attack Success Rate

**DOI:** 10.3390/e25111508

**Published:** 2023-10-31

**Authors:** Fangqun Yang, Yisong Wang

**Affiliations:** State Key Laboratory of Public Big Data, College of Computer Science and Technology, Guizhou University, Guiyang 550025, China; gs.yangfq21@gzu.edu.cn

**Keywords:** complex network, robustness, quasi-Monte Carlo, attack success rate

## Abstract

Analyzing the robustness of networks against random failures or malicious attacks is a critical research issue in network science, as it contributes to enhancing the robustness of beneficial networks and effectively dismantling harmful ones. Most studies commonly neglect the impact of the attack success rate (ASR) and assume that attacks on the network will always be successful. However, in real-world scenarios, attacks may not always succeed. This paper proposes a novel robustness measure called Robustness-ASR (RASR), which utilizes mathematical expectations to assess network robustness when considering the ASR of each node. To efficiently compute the RASR for large-scale networks, a parallel algorithm named PRQMC is presented, which leverages randomized quasi-Monte Carlo integration to approximate the RASR with a faster convergence rate. Additionally, a new attack strategy named HBnnsAGP is introduced to better assess the lower bound of network RASR. Finally, the experimental results on six representative real-world complex networks demonstrate the effectiveness of the proposed methods compared with the state-of-the-art baselines.

## 1. Introduction

Complex networks are powerful representations of various real-world systems, including the Internet, social networks, and power grids. Most networks provide benefits and yield positive effects. However, some networks can also produce negative effects, with the most important examples being terrorism [1] and disease transmission networks [2]. Whether beneficial or harmful, these networks substantially influence the functioning and development of our society. In recent decades, the study of diverse complex networks has gained significant attention from researchers across various fields, such as computer science, statistical physics, systems engineering, and applied mathematics [3,4,5,6,7]. One hot topic in these studies is the error and attack tolerance of complex networks [8,9,10,11,12,13,14,15], a concept referred to as *robustness* within the context of this paper.

The robustness of a network refers to its ability to keep functioning when some of its components, such as nodes or edges, malfunction due to random failures or malicious attacks [12,16,17]. The study of network robustness is valuable from two primary perspectives. Firstly, the failure of components can lead to the breakdown of beneficial networks and result in significant economic losses. A typical example is the Northeast blackout of 2003 [18,19]. Analyzing network robustness aids in developing methods to enhance it. On the other hand, for harmful networks, such as terrorist networks [1] or COVID-19 transmission networks [20], analyzing their robustness assists in developing effective attack strategies to dismantle them. Therefore, analyzing network robustness is of great importance.

To assess the robustness of the network, it is crucial to select an appropriate metric. Since almost all network applications are typically designed to operate in a connected environment [21], network connectivity is selected as the primary indicator to assess network robustness in this study.

The robustness of a network depends not only on its structural features but also on the mechanisms of random failures or malicious attacks. In random failures, nodes or edges are attacked with equal probability. In contrast, malicious attacks carefully select nodes or edges in the network for removal in order to maximally disrupt network functionality. Typically, random failures are less severe than malicious attacks [22]. Therefore, this paper primarily focuses on the latter. Evaluating the impacts of node or edge removal using various malicious attack strategies is a crucial approach to analyzing network robustness. Determining the lower bound of network robustness is critical, as it allows for analysis of network robustness under worst-case scenarios, identification of the most vulnerable components, and development of robustness improvement methods. An effective approach to addressing this issue involves identifying an optimal attack strategy that inflicts maximum damage on the network [23].

Extensive research has been conducted on the robustness of complex networks. Albert et al. [8] studied the robustness of scale-free networks and found that, while these networks are robust to random failures, they are extremely vulnerable to malicious attacks. Iyer et al. [9] conducted a systematic examination of the robustness of complex networks by employing simultaneous and sequential targeted attacks based on various centrality measures such as degree, betweenness, closeness, and eigenvector centrality. Fan et al. [10] proposed a deep reinforcement learning algorithm, FINDER, to effectively identify critical network nodes. Wang et al. [11] introduced region centrality and proposed an efficient network disintegration strategy based on this concept, which combines topological properties and geographic structure in complex networks. Ma et al. [12] conducted a study on the robustness of complex networks against incomplete information. They employed link prediction methods to restore missing network topology information and identify critical nodes. Lou et al. [14] introduced LFR-CNN, a CNN-based approach that utilizes learning feature representation for predicting network robustness, which exhibits excellent predictive performance, including notably smaller prediction errors.

**Figure 1:**
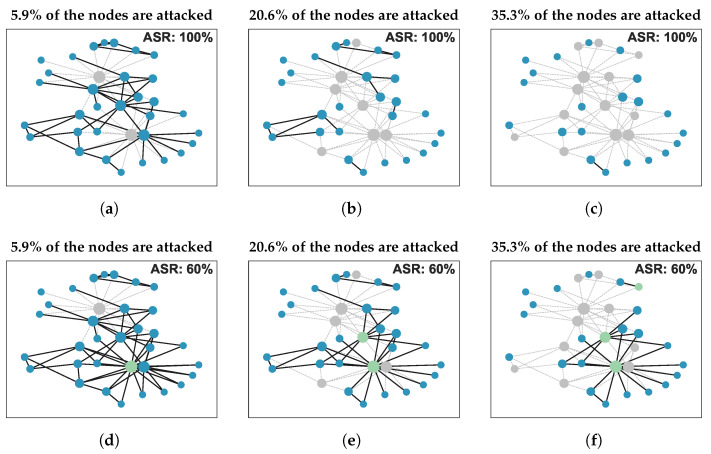
An example of network disintegration processes under different ASR. Gray nodes indicate successful attacks, green nodes represent unsuccessful attacks, and blue nodes denote unattacked nodes. (**a**–**f**) represent scenarios where 5.9%, 20.6%, and 35.3% of network nodes are attacked, with ASR of 100% and 60% respectively.

However, the aforementioned research generally assumes that attacks on the network will always be successful, neglecting the important factor of attack success rate (ASR). In fact, attacks may not succeed in real-world scenarios. For example, even if enemy forces launch an attack on a target within a military communication network, there is no guarantee of successfully destroying it. Figure 1 illustrates the main process of network disintegration under varying ASRs. Moreover, selecting an optimum attack strategy that can lead to maximal destructiveness to the network is challenging due to the NP-hard nature of this problem [10]. Existing methods often encounter difficulties in achieving a desirable balance between effectiveness and computational efficiency.

Therefore, the purpose of this paper is to analyze network robustness when considering ASR under an optimal attack strategy. To achieve this purpose, a novel robustness measure called Robustness-ASR (RASR) is introduced, which utilizes mathematical expectations to evaluate network robustness when considering ASR. In addition, an efficient algorithm called PRQMC is proposed to calculate the RASR for large-scale networks. Furthermore, to assess the lower bound of network RASR, a new attack strategy called HBnnsAGP is proposed. The main contributions of this study are as follows:

- We introduce and define a novel robustness measure called RASR, which utilizes mathematical expectations to assess network robustness when considering the ASR of each node.
- To efficiently calculate the RASR for large-scale networks, we propose the PRQMC algorithm. PRQMC leverages randomized quasi-Monte Carlo (QMC) integration to approximate the RASR with a faster convergence rate and utilizes parallelization to speed up the calculation.
- To assess the lower bound of network RASR, we present a new attack strategy called HBnnsAGP. In HBnnsAGP, a novel centrality measure called BCnns is proposed to quantify the importance of a node.
- The experimental results for six representative real-world networks demonstrate the effectiveness of the proposed methods compared with the baselines.

The rest of this paper is organized as follows. Section 2 provides an introduction to the preliminaries, including classical centrality measures, traditional network robustness measures, and the principles of Monte Carlo (MC) and QMC integration. Section 3 presents the proposed methods for analyzing network robustness when considering ASR, including the RASR, the PRQMC algorithm, and the HBnnsAGP attack strategy. The experiments and results are demonstrated in Section 4. Finally, Section 5 concludes the paper.

## 2. Preliminaries

A complex network can be modeled as an unweighted, undirected graph G=(V,E), where $V(\left|V\right|=N)$ and $E(\left|E\right|=M)$ represent the set of nodes and the set of edges in network *G*, respectively. Network *G* can be also represented as adjacency matrix $A={\left(a_{ij}\right)}_{N\times N}$; if node *i* and node *j* are connected, $a_{ij}=1$, otherwise $a_{ij}=0$.

### 2.1. Centrality Measures

The concept of a centrality measure attempts to quantify how important a node is [24]. Here we introduce two classical centrality measures: degree centrality and betweenness centrality.

#### 2.1.1. Degree Centrality (DC)

DC is the simplest measure of centrality. The DC of a node is defined by its degree, that is, its number of edges. The DC is formally defined as follows.

**Definition** **1.** *Given a network G=(V,E), $A={\left(a_{ij}\right)}_{N\times N}$ is the adjacency matrix of the network G. The DC of node i is defined as:*

(1) $$DC\left(i\right)=\sum_{\begin{array}{c} j\in V \end{array}}a_{ij}.\;$$

The DC is frequently a reliable and effective measure of a node’s importance. A higher DC value typically signifies a more critical node.

#### 2.1.2. Betweenness Centrality (BC)

BC quantifies the number of shortest paths passing through a particular node in a network [25]. BC characterizes the extent to which a node acts as a mediator among all other nodes in a network. Nodes that lie on numerous shortest paths are likely to play a crucial role in information transmission, exhibiting higher BC values. The BC is defined as follows.

**Definition** **2.** *Given a network G=(V,E), the BC of node v in G is defined as:*

(2) $$BC\left(v\right)=\sum_{\begin{array}{c} s,t\in V \end{array}}\frac{\sigma (s,t|v)}{\sigma (s,t)},\;$$

*where v∈V, σ(s,t) is the total number of shortest paths from node s to node t, and σ(s,t∣v) is the number of those paths that pass through node v. σ(s,t)=1 if s=t. σ(s,t∣v)=0 if v∈s,t.*

### 2.2. Accumulated Normalized Connectivity

Traditionally, network robustness has been evaluated by calculating the size of the giant connected component (GCC) after the network has endured attacks. The accumulated normalized connectivity (ANC), also known as *R*, is a well-known measure of network robustness for node attacks [10,16,26]. The ANC is defined as follows.

**Definition** **3.** *For a network G=(V,E), $\left|V\right|=N$. Given an attack sequence of nodes $\left(v_{1},v_{2},\ldots ,v_{N}\right)$, where $v_{i}\in V$ indicates the ith node to be attacked, the ANC of G under this attack sequence is defined as:*

(3) $$ANC\left(v_{1},v_{2},\ldots ,v_{N}\right)=\frac{1}{N}\sum_{k=1}^{N}\frac{\sigma_{gcc}\left(G\backslash \left\{v_{1},v_{2},\ldots ,v_{k}\right\}\right)}{\sigma_{gcc}\left(G\right)},\;$$

*where $\sigma_{gcc}\left(G\backslash \left\{v_{1},v_{2},\ldots ,v_{k}\right\}\right)$ is the size of the GCC of the residual network after the sequential removal of nodes from the set $\left\{v_{1},v_{2},\ldots ,v_{k}\right\}$ in G, and $\sigma_{gcc}\left(G\right)$ is the initial size of the GCC of G before any nodes are removed. The normalization factor $\frac{1}{N}$ ensures that the robustness of networks with different sizes can be compared.*

A larger ANC value indicates a higher level of network robustness against attacks. Additionally, the ANC can be used to assess the destructiveness of attacks, as lower ANC values correspond to more destructive attack strategies. The ANC value can be viewed as an estimate of the area beneath the ANC curve, which is plotted with the horizontal axis as k/N and the vertical axis as $\sigma_{gcc}\left(G\backslash \left\{v_{1},v_{2},\ldots ,v_{k}\right\}\right)/\sigma_{gcc}\left(G\right)$.

### 2.3. Monte Carlo Integration

Monte Carlo (MC) integration is a numerical technique that is particularly useful for higher-dimensional integrals [27]. Caflisch [28] provides a comprehensive review of this method. The integral of a Lebesgue integrable function f(X)  can be expressed as the average or expectation of the function evaluated at random locations. Considering X as a random variable uniformly distributed on the one-dimensional unit interval [0,1], the integration of f(X) over this interval can be represented as follows:

(4) $$I\left[f\right]=E\left[f\left(X\right)\right]=\int_{\left[0,1\right]}f(X)dP(X),\;$$

in which P(X) is the probability measure of X on the interval [0,1], then

(5) dP(X)=dX,

therefore

(6) $$I\left[f\right]=E\left[f\left(X\right)\right]=\int_{\left[0,1\right]}f(X)dX.\;$$

Similarly, for an integral on the unit hypercube ${\left[0,1\right]}^{N}$ in *N* dimensions,

(7) $$I\left[f\right]=E\left[f\left(X\right)\right]=\int_{{\left[0,1\right]}^{N}}f(X)dX,\;$$

in which $X=(x_{1},x_{2},\ldots ,x_{N})$ is a uniformly distributed vector in ${\left[0,1\right]}^{N}$, where $x_{i}\in \left[0,1\right],i\in \left\{1,2,\ldots ,N\right\}$. Given that the hyper-volume of ${\left[0,1\right]}^{N}$ is equal to 1, ${\left[0,1\right]}^{N}$ can be viewed as the total probability space.

The MC integration method approximates definite integrals utilizing random sampling. It draws *K* uniform samples from ${\left[0,1\right]}^{N}$, in turn generating point set $\left\{X_{1},X_{2},\ldots ,X_{K}\right\}$. The empirical approximation of the integral I[f] is then procured by computing the mean of the *K* sample outcomes $f(X_{i})$, which can be expressed as follows:

(8) $$I\left[f\right]\approx I_{K}\left[f\right]=\frac{1}{K}\sum_{i=1}^{K}f(X_{i}).\;$$

According to the Strong Law of Large Numbers [29], this approximation is convergent with probability 1; that is,

(9) $$\lim_{K\to \infty }P\left(\left|I_{K}\left[f\right]-I\left[f\right]\right|=0\right)=1.\;$$

**Figure 2:**
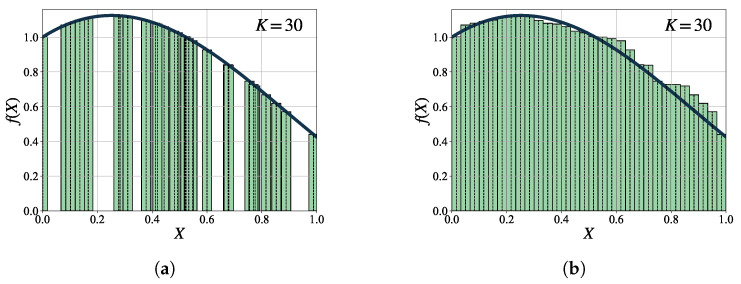
An example of the MC integration method for approximating a definite integral over a one-dimensional unit interval. (**a**) illustrates the approximation of the integral by summing the areas of bars that correspond to the sampled points. Each bar’s height represents the value of f(X) at $X_{i}$, and its width is 1/K, where *K* denotes the total number of samples. (**b**) demonstrates the sequential rearrangement of the bars to prevent overlapping on the X-axis, ensuring a clear visualization of the areas.

Figure 2 illustrates the application of the MC integration method in approximating definite integrals over a one-dimensional unit interval. As shown in Figure 2a, MC integration approximates the area under the curve of the integral by summing the areas of the bars corresponding to the sampled points. The bars are rearranged sequentially to avoid overlap on the X-axis, as shown in Figure 2b.

The error of MC integration is:

(10) $$\varepsilon_{K}=\left|I_{K}\left[f\right]-I\left[f\right]\right|.\;$$

By the Central Limit Theorem [29], for any a,b where a<b, we have:

(11) $$\begin{array}{c} \lim_{K\to \infty }P\left(a<\frac{\varepsilon_{K}}{\sigma /\sqrt{K}}<b\right)=\int_{a}^{b}\frac{1}{\sqrt{2\pi }}e^{-t^{2}/2}dt=P\left(a<v<b\right),\; \end{array}$$

where *v* is a standard normal random variable, and σ is the square root of the variance of *f*, given by

(12) $$\sigma ={\left(\int_{{\left[0,1\right]}^{N}}{\left(f\left(X\right)-I\left[f\right]\right)}^{2}dX\right)}^{1/2}.\;$$

When *K* is sufficiently large, we have:

(13) $$\varepsilon_{K}\approx \sigma K^{-1/2}v.\;$$

This implies that the order of error convergence rate of the MC integration is $O(K^{-1/2})$ [30], which means that the accuracy of the integral error decreases at a rate proportional to the total number of samples as *K* increases. That is, “an additional factor of 4 increase in computational effort only provides an additional factor of 2 improvements in accuracy” [28].

In practical applications, the MC integration method draws *K* uniform samples from an *N*-dimensional pseudo-random sequence (PRS) generated by a computer to obtain the point set $\left\{X_{1},X_{2},\ldots ,X_{K}\right\}$.

### 2.4. Quasi-Monte Carlo Integration

The quasi-Monte Carlo (QMC) integration is a method of numerical integration that operates in the same way as MC integration, but instead uses a deterministic low-discrepancy sequence (LDS) [31] to approximate the integral. The advantage of using LDS is a faster rate of convergence. QMC integration has a rate of convergence close to $O(K^{-1})$, which is much faster than the rate for the MC integration, $O(K^{-1/2})$ [32].

Using the QMC integration method for approximating definite integrals is similar to the MC integration method. This can be expressed as:

(14) $$\begin{array}{c} I\left[f\right]=\int_{{\left[0,1\right]}^{N}}f(X)dX\approx \frac{1}{K}\sum_{i=1}^{K}f(Y_{i}),\; \end{array}$$

where $\left\{Y_{1},Y_{2},\ldots ,Y_{K}\right\}$ is a point set obtained by combining the first *K* points from an *N*-dimensional LDS. Each $Y_{i}$ is an *N*-dimensional point, with $Y_{i}=\left(y_{1}^{\left\{i\right\}},y_{2}^{\left\{i\right\}},\ldots ,y_{N}^{\left\{i\right\}}\right)$ for i∈{1,2,…,K}, and $y_{j}^{\left\{i\right\}}\in \left[0,1\right]$ for j∈{1,2,…,N}.

The error order of the QMC integration can be determined by the Koksma–Hlawka inequality [33,34]; that is,

(15) $$\varepsilon_{K}=\left|\int_{{\left[0,1\right]}^{N}}f(X)dX-\frac{1}{K}\sum_{i=1}^{K}f(Y_{i})\right|<V\left(f\right)D_{K}^{*},\;$$

where V(f) is the Hardy–Krause variation of the function *f*, and $D_{K}^{*}$ is the star discrepancy of $\left\{Y_{1},Y_{2},\ldots ,Y_{K}\right\}$, defined as:

(16) $$D_{K}^{*}=\underset{Q\subset {\left[0,1\right]}^{N}}{\sup}\left|\frac{M(Y_{1},Y_{2},\ldots ,Y_{K})}{K}-\lambda_{N}\left(Q\right)\right|,\;$$

where $M(Y_{1},Y_{2},\ldots ,Y_{K})$ is the number of points in $\left\{Y_{1},Y_{2},\ldots ,Y_{K}\right\}$ inside the region *Q*, and $\lambda_{N}\left(Q\right)$ is the Lebesgue measure of region *Q* in the unit hypercube ${\left[0,1\right]}^{N}$. For more detailed information, please refer to [28].

For an *N*-dimensional LDS comprising *K* points, the star discrepancy of the sequence is $O(K^{-1}{\left(\log K\right)}^{N})$. Consequently, for a function *F* with V(F)<∞, a QMC approximation based on this sequence yields a worst-case error bound in (28) converging at a rate of $O(K^{-1}{\left(\log K\right)}^{N})$ [35]. Since logK≪K, the QMC integration convergence rate approaches $O(K^{-1})$ for low-dimensional cases [30], which is asymptotically superior to MC.

**Figure 3:**
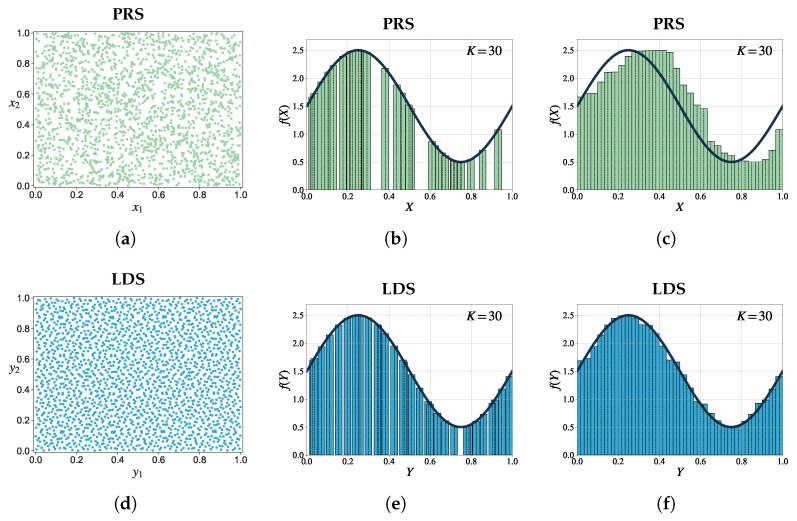
A comparison of MC and QMC integration methods. (**a**,**d**) show the two-dimensional projections of a PRS and an LDS (a Sobol sequence), respectively. (**b**,**c**) depict the MC integration for approximating a definite integral over a one-dimensional unit interval, while (**e**,**f**) present the QMC integration for approximating a definite integral over a one-dimensional unit interval.

Figure 3 illustrates the clear differences between MC and QMC integration methods. The subfigures provide a visual representation of their respective point distributions and demonstrate their application for approximating definite integrals over a one-dimensional unit interval. The points generated from an LDS exhibit greater uniformity than the points generated by a PRS. Consequently, with the same number of sampling points, LDS has the ability to uniformly fill the integration space, resulting in a faster convergence rate.

## 3. Methods

In this section, we first introduce the major problem we focus on in this paper. Then, we give the details of the proposed methods for analyzing network robustness when considering ASR, including the RASR, the PRQMC algorithm, and the HBnnsAGP attack strategy.

### 3.1. Problem Formalization

Typically, it is assumed that removing a node will also remove all of its connected edges. Therefore, in this paper, we only consider node attack strategies.

For a network G=(V,E), $\left|V\right|=N$. A node attack strategy can be represented as a sequence $Seq=(v_{1},v_{2},\ldots ,v_{N})$, where $v_{i}\in V$ indicates the *i*th node to be attacked. Given a predefined metric Φ(Seq) to measure network robustness against attacks, the primary goal is to evaluate the lower bound of network robustness. Therefore, the objective is to minimize Φ(Seq), as presented below:

(17) MinimizeΦ(Seq).

To achieve this objective, it is crucial to determine the optimal node attack strategy that will minimize the Φ(Seq).

### 3.2. The Proposed Robustness Measure RASR

The ANC, as defined in Definition 3, does not consider the ASR, or it is a special case where the ASR of each node is 100%. To this end, the proposed robustness measure RASR utilizes mathematical expectations to assess network robustness when considering ASR. Before introducing the RASR, we first present a weighted ANC (called ANCw), which takes into account both the state of the attack sequence and the associated attack cost.

For a network G=(V,E) with *N* nodes, $Seq=(v_{1},v_{2},\ldots ,v_{N})$ is an attack sequence, where $v_{i}\in V$. The state of Seq is denoted as a random variable $S=(s_{v_{1}},s_{v_{2}},\ldots ,s_{v_{N}})$, where

(18) $$s_{v_{i}}=\left\{\begin{array}{cc} T, & \mathrm{if}\;\mathrm{the}\;\mathrm{attack}\;\mathrm{on}\;v_{i}\;\mathrm{succeeded}\\ F, & \mathrm{otherwise} \end{array}\right..\;$$

Then, the ANCw is defined as follows.

**Definition** **4.** *The ANCw of G under an attack sequence Seq is defined as:*

(19) $$\begin{array}{c} ANCw\left(Seq,S\right)=\frac{1}{N\;+\;1}\sum_{k=0}^{N}\frac{\sigma_{gcc}\left(G\backslash \left\{v_{i}|s_{v_{i}}=T,i=1,2,\cdots ,k\right\}\right)}{\sigma_{gcc}\left(G\right)}\varphi \left(v_{k}\right),\; \end{array}$$

*where $\sigma_{gcc}$ is the same as defined in Definition 3. When k = 0, it indicates that no nodes have been attacked. $\varphi (v_{k})$ is a weighted function; that is,*

(20) $$\varphi \left(v_{k}\right)=\left\{\begin{array}{cc} 0, & \mathrm{if}\;v_{k}\;\mathrm{is}\;\mathrm{an}\;\mathrm{isolated}\;\mathrm{node}\\ 1, & \mathrm{otherwise} \end{array}\right..\;$$

There are two main reasons for using the weighted function $\varphi (v_{k})$. Firstly, it is important for an attacker to choose an optimal attack strategy at a minimum attack cost to efficiently disintegrate the network [11,23]. Secondly, as illustrated in Figure 1, with an increased number of nodes removed, the network will eventually fragment into isolated nodes, thereby losing its functionality as a network. Therefore, this paper sets the attack cost of an isolated node to 0.

Let $P_{v}=\left(p_{v_{1}},p_{v_{2}},\ldots ,p_{v_{N}}\right)$ represent the ASR of each node corresponding to Seq, where $p_{v_{i}}$ represents the ASR of node $v_{i}$. Assuming that attacks on different nodes are independent, then the probability of S is

(21) $$p\left(S\right)=\prod_{i=1}^{N}p\left(s_{v_{i}}\right),\;$$

where

(22) $$p\left(s_{v_{i}}\right)=\left\{\begin{array}{cc} p_{v_{i}}, & \mathrm{if}\;s_{v_{i}}=T\\ 1-p_{v_{i}}, & \mathrm{otherwise} \end{array}\right..\;$$

Based on the above formulas, the proposed RASR can be defined as follows.

**Definition** **5.** *Considering the ASR of each node, the robustness of a network G against an attack sequence Seq can be quantified by the RASR, which is defined as:*

(23) $$\begin{array}{c} RASR=E\left(ANCw\left(Seq,S\right)\right)=\sum_{S\in \Omega }ANCw(Seq,S)p(S) \end{array},\;$$

*where S is a random variable representing the state of Seq, *Ω* is the sample space of S, and E(ANCw(Seq,S)) is the expectation of the ANCw.*

In theory, the value of RASR can be calculated using (23) once all the samples of S are obtained in the sample space Ω. However, it confronts “the curse of dimensionality” [36] when applied to networks with a large number of nodes. In such cases, the size of Ω grows exponentially to $2^{N}$. As a result, the analytical approach becomes infeasible when *N* is significantly large.

### 3.3. The Proposed PRQMC Algorithm

To efficiently calculate the RASR for large-scale networks, the PRQMC algorithm is proposed, which leverages randomized QMC integration to approximate the RASR with a faster convergence rate and utilizes parallelization techniques to speed up the calculation. In the following, we first introduce the RASR calculation model based on QMC integration and then give the PRQMC algorithm.

#### 3.3.1. RASR Calculation Model Based on QMC Integration

The RASR of a network *G*, as defined in Definition 5, can be expressed using Lebesgue integration based on the principle of MC integration (see Section 2); that is,

(24) $$\begin{array}{c} RASR=E\left(ANCw\left(Seq,S\right)\right)=\int_{\Omega }ANCw(Seq,S)dP(S),\; \end{array}$$

where $S=(s_{v_{1}},s_{v_{2}},\ldots ,s_{v_{N}})$ denotes a random variable representing the state of an attacking sequence Seq, Ω is the sample space of S, and P(S) is the probability measure of S.

Let $P_{v}=\left(p_{v_{1}},p_{v_{2}},\ldots ,p_{v_{N}}\right)$ represent the ASR of each node corresponding to Seq, and let $X=(x_{1},x_{2},\ldots x_{N})$ be a uniformly distributed vector in ${\left[0,1\right]}^{N}$, where $x_{i}\in \left[0,1\right],i\in \left\{1,2,\ldots ,N\right\}$. Then, $S=(s_{v_{1}},s_{v_{2}},\ldots ,s_{v_{N}})$ can be represented as follows:

(25) S=G(X),

where

(26) $$s_{v_{i}}=G_{i}\left(x_{i}\right)=\left\{\begin{array}{cc} T, & \mathrm{if}\;x_{i}\leq p_{v_{i}}\\ F, & \mathrm{otherwise} \end{array}\right.,i\in \left\{1,2,\ldots ,N\right\}.\;$$

When the Seq is determined, then ANCw(Seq,S) can be represented as a function of X; that is,

(27) F(X)=ANCw(Seq,G(X))=ANCw(Seq,S).

By substituting (27) into (24) and transforming the integral space from Ω to ${\left[0,1\right]}^{N}$, we obtain the following expression for RASR:

(28) $$RASR=E\left[F\left(X\right)\right]=\int_{{\left[0,1\right]}^{N}}F(X)dP(S).\;$$

This equation represents the integration of F(X) with respect to the probability measure P(S) over the *N*-dimensional unit hypercube ${\left[0,1\right]}^{N}$.

**Figure 4:**
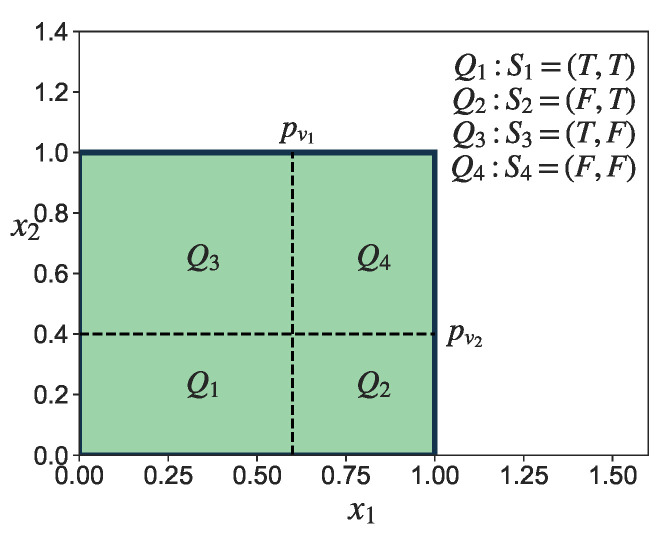
An example to illustrate the division of the unit hypercube, where N=2 and $P_{v}=\left(p_{v_{1}},p_{v_{2}}\right)$. The unit hypercube ${\left[0,1\right]}^{2}$ is divided into 4 regions, namely $Q_{1},Q_{2},Q_{3},Q_{4}$, where each region corresponds to a state of Seq, denoted by $S_{1},S_{2},S_{3},S_{4}$.

For the given network *G*, the sample space Ω has a size of $2^{N}$. Let the state of Seq be $S_{i}$, where $i\in \{1,2,3,\ldots ,2^{N}\}$. Based on $P_{v}$, the unit hypercube ${\left[0,1\right]}^{N}$ can be divided into $2^{N}$ regions denoted by $Q_{i}$, where region $Q_{i}$ corresponds to state $S_{i}$, $i\in \{1,2,\ldots 2^{N}\}$. Figure 4 illustrates this process for the case when N=2. Then, the integral in (28) can be transformed into:

(29) $$\int_{{\left[0,1\right]}^{N}}F(X)dP(S)=\sum_{i=1}^{2^{N}}\int_{Q_{i}}F(X^{i})dP\left(S_{i}\right),\;$$

where $X^{\left\{i\right\}}$ is a vector uniformly distributed within region $Q_{i}$.

The Lebesgue measure of region $Q_{i}$ in ${\left[0,1\right]}^{N}$, denoted by $\lambda_{N}\left(Q_{i}\right)$, is equivalent to the probability measure of $S_{i}$, denoted as $P(S_{i})$. Based on the principle of MC integration, we have:

(30) $$\begin{array}{c} \sum_{i=1}^{2^{N}}\int_{Q_{i}}F(X^{\left\{i\right\}})dP\left(S_{i}\right)=\sum_{i=1}^{2^{N}}\int_{Q_{i}}F(X^{\left\{i\right\}})dX^{\left\{i\right\}}=\int_{{\left[0,1\right]}^{N}}F(X)dX.\; \end{array}$$

Combining (28), (29), and (30), we obtain:

(31) $$RASR=E\left[F\left(X\right)\right]=\int_{{\left[0,1\right]}^{N}}F(X)dX.\;$$

By referencing (14) and (31), the RASR of a network can be approximated using the QMC integration method. The approximation of RASR, denoted by $\hat{R}$, is defined as follows.

**Definition** **6.** *Consider a network G=(V,E) with N nodes. Suppose a sequence of nodes $Seq=(v_{1},v_{2},\ldots ,v_{N})$ is targeted for attack, and $P_{v}=\left(p_{v_{1}},p_{v_{2}},\ldots ,p_{v_{N}}\right)$ signifies the ASR of each node. The RASR of the network G can be approximated by $\hat{R}$, which is defined as:*

(32) $$\begin{array}{c} \hat{R}=\frac{1}{K}\sum_{i=1}^{K}F(Y_{i})\approx RASR. \end{array}$$

*Here, $\left\{Y_{1},Y_{2},\ldots ,Y_{K}\right\}$, as specified in (14), represents a set of points obtained from an N-dimensional LDS. K is the total number of samples. The function F(X) is defined in (27).*

The error bound of the QMC integral is determined by the star discrepancy of the chosen LDS, making the selection of LDS important for improving the accuracy of approximations. Two frequently used LDSs are the Halton sequence and the Sobol sequence [37]. In this research, the Sobol sequence is adopted, as it demonstrates better performance in higher dimensions compared to the Halton sequence [38].

#### 3.3.2. Parallel Randomized QMC (PRQMC) Algorithm

Despite the faster convergence rate of the QMC integration method compared to MC integration, it still necessitates a large number of samples to calculate the average value. Furthermore, the calculation of function ANCw(Seq,S), typically done through attack simulations, demands considerable computational resources, especially for large-scale networks [39]. Consequently, the computational process of obtaining $\hat{R}$ for large-scale networks remains time-consuming. Additionally, due to the deterministic nature of the LDS, the QMC integration method can be seen as a deterministic algorithm, thus presenting challenges in assessing the reliability of numerical integration results and potentially leading to being stuck in local optima. In light of these issues, the PRQMC algorithm capitalizes on the benefits of the Randomized QMC method and parallelization.

The PRQMC algorithm improves computational efficiency through parallelization. This is because the computational cost of sampling the attack sequence’s state S is significantly lower than that of computing the function ANCw(Seq,S). Therefore, by initially sampling the attack sequence’s state S and obtaining a sufficient number of samples, it is possible to calculate the $\hat{R}$ by parallelizing the computation of the function ANCw(Seq,S) with various samples. This approach effectively accelerates the calculation process by distributing the task across multiple processors or computing nodes.

Additionally, the PRQMC algorithm enhances randomness by randomly sampling points from the LDS, providing unbiased estimation and improved variance reduction capabilities. This is particularly advantageous in high-dimensional problems, where RQMC often outperforms QMC in terms of accuracy and efficiency [40].

The procedure of the PRQMC algorithm is presented in Algorithm 1, which consists of two main steps: “sampling stage” and “paralleling stage”. In the sampling stage, we first randomly sample *K* points $\left\{Y_{1},Y_{2},\ldots ,Y_{K}\right\}$ from an *N*-dimensional Sobol sequence, then determine *K* states of the attack sequence, $\left\{S_{1},S_{2},\ldots ,S_{K}\right\}$, by comparing the values of each dimension of the sampled points with the ASR of each node. In the paralleling stage, we parallelize the computation of the function $ANCw(Seq,S_{i})$, then obtain $\hat{R}$ by calculating the average value of $ANCw(Seq,S_{i})$.
**Algorithm 1**  PRQMC(G,Seq,P,K)**Input:** G=(V,E): a network with *N* nodes,
           $Seq=(v_{1},v_{2},\ldots ,v_{N})$: an attacking sequence of *G*,
           $P=(p_{v_{1}},p_{v_{2}},\ldots ,p_{v_{N}})$: ASR of each node in Seq,
           *K*: the total number of samples.**Output:**  $\hat{R}$: the approximate value of the RASR of *G*.
     **Step 1: Sampling stage.**   1:sampling *K* points $\left\{Y_{1},Y_{2},\ldots ,Y_{K}\right\}$ randomly from an *N*-dimensional Sobol sequence, where $Y_{i}=\left(y_{1}^{\left\{i\right\}},y_{2}^{\left\{i\right\}},\ldots ,y_{N}^{\left\{i\right\}}\right)$ for i∈{1,2,…,K};   2:let $State=\{S_{1},S_{2},\ldots ,S_{K}\}$, where $S_{i}=\left(s_{v_{1}}^{\left\{i\right\}},s_{v_{2}}^{\left\{i\right\}},\ldots ,s_{v_{N}}^{\left\{i\right\}}\right)$ for i∈{1,2,…,K};   3:**for** i=1 to *K* **do**   4:    $s_{v_{j}}^{\left\{i\right\}}=\left\{\begin{array}{c} T,y_{j}^{\left\{i\right\}}\leq p_{v_{j}}\\ F,y_{j}^{\left\{i\right\}}>p_{v_{j}} \end{array}\right.,j\in \left\{1,2,\cdots ,N\right\}$;   5:**end for****Step 2: Paralleling stage.**   6:let $Res=\{{\hat{R}}_{1},{\hat{R}}_{2},\ldots ,{\hat{R}}_{K}\}$;   7:**parallel for all **$S_{i}\in State$ **do**   8:    ${\hat{R}}_{i}=ANCw\left(Seq,S_{i}\right)$;   9:**end for**  10:$\hat{R}=\frac{1}{K}\sum_{i=1}^{K}{\hat{R}}_{i}$; 11:**return** $\hat{R}$.

### 3.4. The Proposed HBnnsAGP Attack Strategy

To assess the lower bound of network RASR, a new attack strategy called High BCnns Adaptive GCC-Priority (HBnnsAGP) is presented. In HBnnsAGP, a novel centrality measure called BCnns is proposed to quantify the significance of a node, and the GCC-priority attack strategy is utilized to improve attack effectiveness. Algorithm 2 describes the procedure of HBnnsAGP, which contains two steps: “obtaining the first part of Seq” and “obtaining the second part of Seq”. In the first step, the algorithm obtains the first part of the attack sequence by iteratively removing the node with the highest BCnns in GCC and recalculating BCnns for the remaining nodes until only isolated nodes remain in the residual network. In the second step, the algorithm arranges these isolated nodes in descending order according to their DC values in the initial network to obtain the second part of the attack sequence. This procedure is aimed at improving the effectiveness of attacks when the ASR is below 100%. It is important to note that isolated nodes when the ASR is 100% may no longer remain isolated, as shown in Figure 1. Additionally, previous research has shown that there is minimal difference in destructiveness between simultaneous attacks and sequential attacks based on DC [9]. Therefore, by sorting these isolated nodes in descending order based on their DC values from the initial network (similar to the approach used in simultaneous attacks), the second step further improves the effectiveness of attacks when the ASR is less than 100%.
**Algorithm 2** HBnnsAGP($G,N_{1},N_{2}$)**Input:** G=(V,E): a network with *N* nodes,$N_{1}$ and $N_{2}$: sampling numbers.**Output:** Seq: an attacking sequence of *G*.   1:let Seq be an empty list;   2:$G_{0}=\left(V_{0},E_{0}\right)\leftarrow G$;**Step 1: Obtaining the first part of Seq.**   3:**while** E≠∅ **do**   4:    $G_{c}=\left(V_{c},E_{c}\right)\leftarrow$ get the GCC of *G*;   5:    S,T←SelectST($G_{c},N_{1},N_{2}$)   6:    **for all** $v\in V_{c}$ **do**   7:          $BC_{\mathrm{nns}}\left(v\right)=\sum_{\begin{array}{c} s\in S,t\in T \end{array}}\frac{\sigma (s,t|v)}{\sigma (s,t)}$;   8:    **end for**   9:    $attack\_node\leftarrow \underset{v\in V_{c}}{\arg\max}\left(BC_{\mathrm{nns}}\left(v\right)\right)$; 10:    append attack_node to the end of Seq; 11:    G←G\{attack_node}; 12:**end while****Step 2: Obtaining the second part of Seq.** 13:$V_{r}\leftarrow \left(V_{0}\backslash Seq\right)$; 14:sort the nodes of $V_{r}$ decreasing by DC values from $G_{0}$; 15:$Seq\leftarrow Seq+V_{r}$; 16:**return** Seq.

In the following, we first introduce the BCnns and then give the GCC-priority attack strategy.

#### 3.4.1. Non-Central Node Sampling Betweenness Centrality (BCnns)

Contrasted with BC (see Definition 2), which evaluates a node’s role as a mediator in the network based on the count of shortest paths it traverses for all node pairs, BCnns quantifies the importance of nodes acting as bridge nodes between different network communities by counting the number of shortest paths that pass through a node for specific pairs of non-central nodes (nodes located on the periphery of the network and with less importance). These bridge nodes typically serve as mediators for non-central nodes across different communities. The BCnns is defined as follows.

**Definition** **7.** *For a network G=(V,E) with N nodes, the BCnns of node v in network G is:*

(33) $$\begin{array}{c} BC_{\mathrm{nns}}\left(v\right)=\sum_{\begin{array}{c} s\in S,t\in T \end{array}}\frac{\sigma (s,t|v)}{\sigma (s,t)},\; \end{array}$$

*where $S,T\subset V_{nns}$, $V_{nns}$ is the set of non-central nodes sampled from V, $V_{nns}\subset V$, and S∩T=∅. The σ(s,t) and σ(s,t∣v) have the same meaning as in Definition 2.*

By selecting the appropriate pairs of non-central nodes, BCnns can more effectively measure the significance of nodes as bridges between different communities in a network. While these bridge nodes may not have the highest BC value, they are crucial for maintaining overall network connectivity and could potentially have the highest BCnns value.

The definition of BCnns highlights the importance of selecting suitable nodes for sets *S* and *T*. Thus, we proposed an algorithm called SelectionST for node selection. Algorithm 3 describes the procedure of SelectionST. Initially, the nodes are sorted in ascending order based on their DC values, and the first $N_{1}$ nodes with lower DC values are selected to create the non-central node set $V_{nns}$. This is because nodes with lower DC values typically have lower centrality and are considered non-central nodes. Next, in order to achieve a more balanced sampling, $V_{nns}$ is divided into two subsets: $V_{nns}^{odd}$, containing nodes at odd indices, and $V_{nns}^{even}$, containing nodes at even indices. Lastly, $N_{2}$ nodes are randomly sampled from $V_{nns}^{odd}$ to create set *S*, and $N_{2}$ nodes are similarly sampled from $V_{nns}^{even}$ to form set *T*.
**Algorithm 3** SelectionST($G_{c},N_{1},N_{2}$)**Input:** $G_{c}=\left(V_{c},E_{c}\right)$: the GCC of network *G*,            $N_{1}$ and $N_{2}$: sampling numbers.**Output:**  *S* and *T*: the sets of sampling nodes.
   1:**if** $\left|V_{c}\right|<=N_{1}$ **then**   2:    $N_{1}\leftarrow$$\left\lfloor 0.85*\left|V_{c}\right|\right\rfloor$;   3:**end if**   4:sort the nodes of $V_{c}$ increasing by DC values;   5:$V_{nns}\leftarrow$ choose first $N_{1}$ nodes of $V_{c}$;   6:$V_{nns}^{odd}\leftarrow$ choose nodes at odd indices of $V_{nns}$;   7:$V_{nns}^{even}\leftarrow$ choose nodes at even indices of $V_{nns}$;   8:$N_{2}\leftarrow$ Min($\left|V_{nns}^{odd}\right|$, $\left|V_{nns}^{even}\right|,N_{2}$)   9:S← choose $N_{2}$ nodes of $V_{nns}^{odd}$ randomly; 10:T← choose $N_{2}$ nodes of $V_{nns}^{even}$ randomly; 11:**return** S,T.


The $N_{1}$ and $N_{2}$ are chosen based on the size of the network and the node degree distribution. Typically, both $N_{1}$ and $N_{2}$ are much smaller compared to the total number of nodes *N*. Therefore, BCnns have higher computational efficiency compared to BC, especially for large-scale networks.

**Figure 5:**
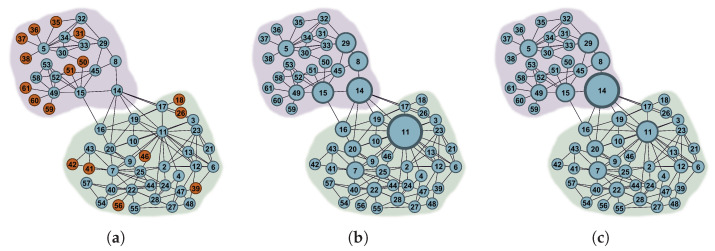
An illustrative example of non-central nodes and comparison of BC and BCnns. In this figure, (**a**) highlights non-central nodes in red, (**b**) showcases node sizes based on BC, and (**c**) showcases node sizes based on BCnns.

Figure 5 demonstrates the differences between BC and BCnns. Specifically, Figure 5a identifies the non-central nodes in red, Figure 5b showcases node sizes based on BC values, and Figure 5c adjusts node sizes based on their BCnns values. Notably, node 14 plays a critical bridging role between two communities, a role that BCnns captures more accurately than BC.

#### 3.4.2. GCC-Priority Attack Strategy

As the attack progresses, the network fragments into connected components of varying sizes. The importance of these components varies within the residual network. The GCC refers to the largest connected component containing the most nodes. The destruction of the GCC accelerates the collapse of the network. The GCC-priority attack strategy enhances the attack’s effectiveness by targeting nodes within the GCC at each stage of the attack process.

## 4. Experimental Studies

In this section, we present a series of experiments to verify the effectiveness of our proposed methods. Firstly, we introduce the experimental settings, including network datasets and baselines. Next, we compare the proposed PRQMC method with the baselines. Additionally, we demonstrate the effectiveness of the proposed HBnnsAGP attack strategy. Finally, we present further discussions of network robustness when considering the ASR.

### 4.1. Experimental Settings

#### 4.1.1. Datasets

In our experiments, we selected six real-world classic complex networks of different scales, including Karate [41], Krebs [10], Airport [42], Crime [42], Power [42], and Oregon1 [43].

1. *Karate*: This is a network depicting relationships in a karate club recorded by Zachary. Nodes represent club members, and each edge connects two members of the club.
2. *Krebs*: The network is associated with the 9/11 attack. The nodes represent the terrorists involved in the network, while the edges depict their communication patterns.
3. *Airport*: This is a network consisting of direct air routes between American airports in 1997. Each node represents an airport, and the edges represent connections between airports.
4. *Crime*: This network represents a criminal network that is derived from a bipartite network of individuals and criminal activities. In this network, each node represents an individual, and an edge connects two individuals involved in the same criminal activity.
5. *Power*: This network represents the high-voltage power grid in the western United States. The nodes represent transformers, substations, and generators, while the edges represent high-voltage transmission lines.
6. *Oregon1*: This network showcases peering information of Autonomous Systems (AS) inferred from Oregon route-views. Each AS is represented by a node, and the edges depict the relationships between the AS.

Table 1 provides a detailed summary of these networks, with *N* and *M* representing the number of nodes and edges, respectively; <*k*> and MaxDeg denote the network’s average degree and maximal degree, respectively; and *C* denotes the average clustering coefficient.

**Figure 6:**
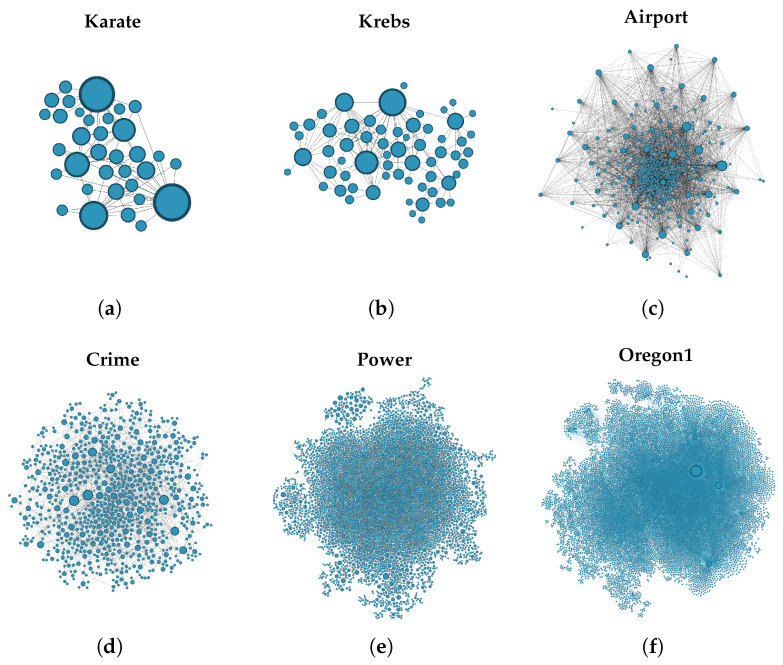
The topologies of six real-world networks. The size of each node is proportional to its degree. (**a**) Karate, (**b**) Krebs, (**c**) Airport, (**d**) Crime, (**e**) Power, (**f**) Oregon1.

The topologies of these networks are shown in Figure 6.

#### 4.1.2. Comparison Methods

To show the effectiveness of the proposed PRQMC algorithm, we compare it with MC and QMC methods.

- MC: This calculates the estimated value of $\hat{R}$ using original MC integration and generates a set of points from a PRS.
- QMC: This calculates the estimated value of $\hat{R}$ using original QMC integration and generates a set of points from an LDS.

To show the effectiveness of the proposed HBnnsAGP attack strategy, we compare it with random failures and three representative baseline attack strategies, including HDA [10], HBA [25], and FINDER [10].

- Random Failures (RF): Nodes are removed from the network in a random order.
- High Degree Adaptive (HDA): HDA is an adaptive version of the high degree method that ranks nodes based on their DC and sequentially removes the node with the highest DC. HDA recomputes the DC of the remaining nodes after each node removal and is recognized for its superior computational efficiency.
- High Betweenness Adaptive (HBA): HBA is an adaptive version of the high betweenness method. It operates by iteratively removing the node with the highest BC and recomputing BC for the remaining nodes. HBA has long been considered the most effective strategy for the network dismantling problem in the node-unweighted scenario [44]. However, the high computing cost prohibits its use in medium- and large-scale networks.
- FINDER: FINDER is notable as an algorithm based on deep reinforcement learning, which achieves superior performances in terms of both effectiveness and efficiency.

We implemented the proposed algorithm and baselines using the Python programming language. All experiments were performed on a server AMD EPYC 7742 64-Core Processor @ 2.25 GHz, with memory (RAM) 1024 GB, running the Linux Ubuntu 11.10 Operating System.

### 4.2. Comparison of the PRQMC with Baselines

This subsection presents the comparison results to demonstrate the effectiveness of the proposed algorithm, PRQMC, on six real-world complex networks. Specifically, we compare PRQMC with two baselines: MC and QMC. All experiments use the same attack strategy, and the ASR of each node is randomly generated.

**Figure 7:**
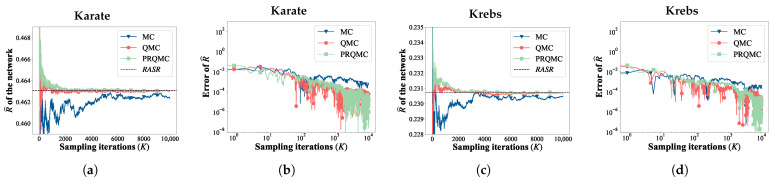
Comparison of the convergence and error of the PRQMC, QMC, and MC methods in assessing robustness for two smaller-scale networks. Convergence and error curves of Karate (**a**,**b**), Krebs (**c**,**d**).

**Figure 8:**
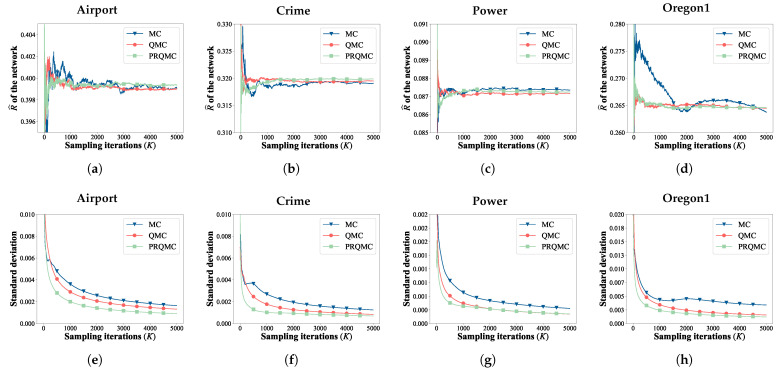
Comparison of the convergence and standard deviation of the PRQMC, QMC, and MC methods in assessing robustness for four larger-scale networks. Convergence and standard deviation curves of Airport (**a**,**e**), Crime (**b**,**f**), Power (**c**,**g**), Oregon1 (**d**,**h**).

We first compare PRQMC with the baselines on two small-scale networks (Karate and Krebs). This is because precise values of RASR can be calculated analytically for small-scale networks. Then, for large-scale networks (Airport, Crime, Power, and Oregon1), we utilize the standard deviation curve as the convergence criterion, as the analytical method is not applicable to large-scale networks. Figure 7 and Figure 8 present the comparison of the convergence and error between PRQMC and baselines. The figure clearly illustrates that PRQMC achieves faster convergence and better accuracy with fewer samples compared to the baselines.

Additionally, Table 2 presents a comparison of the computational efficiency of PRQMC and the baselines, each with 5000 sampling iterations. In the PRQMC method, the number of parallel computing processes is set based on the network size, assigning 25 processes to Karate and Krebs, and 100 processes to the other networks. The results in Table 2 indicate that the PRQMC method outperforms in terms of computational efficiency. Specifically, the PRQMC method operates nearly 50 times faster than the QMC and MC methods on Oregon1.

### 4.3. Comparison of the HBnnsAGP with Baselines

In this subsection, we demonstrate the effectiveness and efficiency of the proposed HBnnsAGP attack strategy. Specifically, we compare HBnnsAGP with HDA, HBA, FINDER, and RF for six real-world complex networks, while considering different ASR conditions. Initially, we employ various strategies to generate corresponding attack sequences. Subsequently, we utilize the PRQMC method to calculate the $\hat{R}$ value under the following ASR distribution scenarios.

1. *ASR = 100%*: The ASR of each node is set to 100%.
2. *ASR = 90%*: The ASR of each node is set to 90%.
3. *ASR = 80%*: The ASR of each node is set to 80%.
4. *ASR = 70%*: The ASR of each node is set to 70%.
5. *ASR = 60%*: The ASR of each node is set to 60%.
6. *ASR = 50%*: The ASR of each node is set to 50%.
7. *ASR = 50% for the first 30% of nodes*: In the attack sequence generated by different attack strategies, the ASR of the first 30% of nodes is set to 50%.
8. *Random ASR*: The ASR of each node is randomly set between 50% and 100%. To obtain more reliable results, the average of 10 experimental outcomes is taken.

The sample numbers ($N_{1}$ and $N_{2}$) for different networks used in HBnnsAGP are presented in Table 3. Table 4 presents the $\hat{R}$ values of networks in the four specified scenarios. The data demonstrate that HBnnsAGP outperforms other attack strategies in terms of destructiveness in the majority of cases. The destructiveness of HBnnsAGP, on average, has increased by 7.01%, 4.05%, 7.62%, and 40.51% compared to FINDER, HBA, HDA, and RF, respectively.

Table 5 presents a comparison of computation times for HBnnsAGP and the baselines. As the network size increases, the computation time for the HBA method becomes excessively long. In contrast, the HBnnsAGP method maintains commendable computational efficiency even for larger-scale networks. For the Oregon1 network, HBnnsAGP is approximately 28 times faster than HBA. While the computational efficiency of HBnnsAGP slightly lags behind that of FINDER and HDA for larger-scale networks, it surpasses them in terms of attack destructiveness.

**Figure 9:**
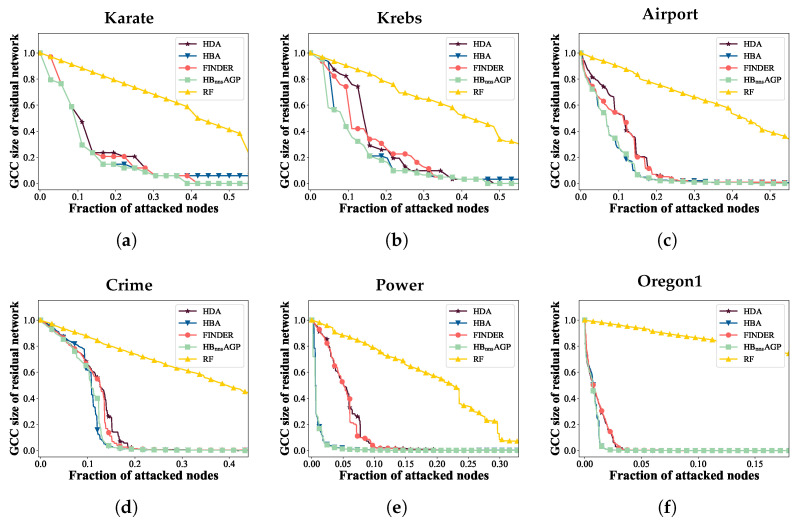
The ANCw curves of networks under different attack strategies: (**a**) Karate, (**b**) Krebs, (**c**) Airport, (**d**) Crime, (**e**) Power, (**f**) Oregon1.

Figure 9 represents the ANCw curves of the networks under various attack strategies when the ASR of each node is set to 100%. In this scenario, the state of the attack sequence is unique. The figure shows that HBnnsAGP excels at identifying critical nodes in the network, leading to the effective disruption of the network structure compared to other methods. Hence, the effectiveness of the proposed HBnnsAGP attack strategy is verified.

### 4.4. Further Discussions about Network Robustness Considering ASR

The role of ASR in determining network robustness is a complex yet critical aspect to consider when assessing the effectiveness of an attack strategy. A higher ASR implies a more successful attack, leading to a greater extent of network disruption. Conversely, a lower ASR indicates a more robust network that can resist the attack without significant damage.

Our analysis, as evidenced by the data presented in Table 4, clearly indicates that a decrease in ASR corresponds to an increase in network robustness. This is due to the fact that nodes with lower ASR are less susceptible to destruction, thereby enhancing the network’s resilience. This trend is consistent across all strategies, including HBnnsAGP, FINDER, HBA, HDA, and RF. Specifically, for every 10% decrease in ASR, the average $\hat{R}$ value for the HBnnsAGP strategy increases by approximately 7–8, indicating a significant improvement in network robustness. This suggests that enhancing node protection to reduce ASR can effectively bolster the robustness of the network. This insight is crucial for designing more robust networks.

Interestingly, we found that enhancing the protection of a small subset of critical nodes, resulting in reduced ASR, can effectively enhance network robustness. This is demonstrated in Scenarios 6 and 7 in Table 4, where merely reducing the ASR of the initial 30% of nodes in the attack sequence (Scenario 7) significantly enhances network robustness. This improvement is approximately 78.25% of that observed in Scenario 6. This highlights the importance of identifying and protecting key nodes within a network. By allocating resources to enhance the protection of these crucial nodes, the robustness of the network can be significantly improved. This strategy is particularly beneficial in scenarios where resources for network protection are limited, thus necessitating prioritized allocation.

However, while ASR is a valuable metric for evaluating network robustness, it is essential to recognize that factors can vary significantly depending on the specific domain. It should be incorporated alongside other network characteristics to achieve a comprehensive evaluation of network robustness. For example, in a power grid network, the failure of a single power station can lead to load redistribution, which can cause overloads and subsequent failures in other parts of the system. This cascading failure can lead to widespread power outages. Thus, improving the protection of individual power stations without considering the overall system dynamics may not substantially enhance the system’s robustness.

Therefore, future research could focus on developing more sophisticated metrics that consider the complexity and specific characteristics of different domains, thus achieving a more accurate and detailed evaluation of network robustness. Furthermore, it would be interesting to explore how to optimally allocate resources to enhance the resilience of critical nodes when resources for network protection are limited.

## 5. Conclusions

In this paper, we conducted a study to analyze the robustness of networks when considering ASR. Firstly, we introduce a novel metric called RASR to assess network robustness in this scenario. Then, we propose the PRQMC algorithm to efficiently calculate the RASR for large-scale networks. PRQMC utilizes RQMC integration to approximate the RASR with a faster convergence rate and employs parallelization to speed up the calculation. Next, we propose a new attack strategy called HBnnsAGP to evaluate the lower bound of network RASR. In HBnnsAGP, we quantify the significance of a node using BCnns and enhance the destructiveness of the attack using the GCC-priority attack strategy. Experimental results on six representative real-world networks demonstrate the effectiveness of the proposed methods. Furthermore, our work demonstrates that reinforcing the protection of a small subset of critical nodes significantly improves network robustness. These findings offer valuable insights for devising more robust networks, especially in scenarios where resources for network protection are limited. The efficiency of the proposed methods can be further enhanced, particularly when analyzing ultra-large-scale networks. In future research, we aim to explore efficient algorithms to enhance the network RASR and devise promising methods for analyzing ultra-large-scale networks.

## Figures and Tables

**Table 1:** Basic information for six real-world networks. *N* and *M* represent the number of nodes and edges, respectively; <*k*> and MaxDeg denote the network’s average degree and maximal degree, respectively; and *C* is the average clustering coefficient.

Network	*N*	*M*	<*k*>	MaxDeg	*C*
Karate [41]	34	78	4.59	17	0.571
Krebs [10]	62	159	5.13	22	0.591
Airport [42]	332	2126	12.81	139	0.625
Crime [42]	829	1473	3.55	25	0.008
Power [42]	4941	6594	2.67	19	0.107
Oregon1 [43]	10,670	22,002	4.12	2312	0.456

**Table 2:** Computational time comparison of PRQMC, QMC, and MC methods (s). Smaller values are better (best in bold).

Network	MC	QMC	PRQMC
Karate	1.8	1.7	**0.4**
Krebs	4.5	4.3	**0.6**
Airport	106.7	104.9	**3.0**
Crime	518.2	520.6	**7.4**
Power	20,525.7	20,529.1	**343.7**
Oregon1	213,748.2	213,758.1	**4262.4**

**Table 3:** The sample numbers ($N_{1}$ and $N_{2}$) for different networks used in HBnnsAGP.

Network	$N_{1}$	$N_{2}$
Karate	16	8
Krebs	30	16
Airport	100	60
Crime	120	80
Power	1300	80
Oregon1	2300	80

**Table 4:** The robustness of networks under different ASR. All $\hat{R}$ values are multiplied by 100. Smaller values represent better attack destructiveness for attack strategies (best in bold).

Scenario	Network	HBnnsAGP	FINDER	HBA	HDA	RF
1. ASR = 100%	Karate	**12.77**	14.12	15.04	15.04	42.86
	Krebs	**12.26**	16.26	14.21	17.23	42.96
	Airport	**7.53**	10.25	7.93	11.10	43.19
	Crime	**9.90**	11.04	10.14	11.54	39.57
	Power	**0.91**	5.02	1.01	5.23	20.29
	Oregon1	**0.68**	1.06	0.73	1.01	36.47
	Avg score	**7.34**	9.63	8.18	10.19	37.56
2. ASR = 90%	Karate	**22.26**	23.92	23.79	24.81	47.25
	Krebs	**20.70**	23.59	21.49	24.40	47.09
	Airport	**20.94**	23.35	22.23	23.74	47.74
	Crime	**16.44**	17.06	17.23	16.95	43.52
	Power	2.41	6.06	**2.37**	6.39	22.51
	Oregon1	**7.30**	9.01	8.18	8.76	40.89
	Avg score	**15.01**	17.17	15.88	17.51	41.50
3. ASR = 80%	Karate	**31.52**	33.00	32.93	34.09	52.25
	Krebs	**28.75**	30.82	29.12	31.37	51.82
	Airport	**31.87**	33.86	33.59	34.17	52.77
	Crime	**23.81**	26.00	25.72	25.43	48.37
	Power	4.09	7.44	**4.04**	7.77	25.44
	Oregon1	**16.71**	19.26	18.41	18.91	46.12
	Avg score	**22.79**	25.06	23.97	25.29	46.13
4. ASR = 70%	Karate	**40.89**	42.1	42.29	43.3	57.75
	Krebs	**37.7**	39.2	37.8	39.44	57.13
	Airport	**41.65**	43.25	43.23	43.51	58.10
	Crime	**34.1**	37.76	37.11	37.3	54.47
	Power	**6.55**	9.65	6.57	9.95	29.24
	Oregon1	**27.37**	30.02	29.36	29.68	51.95
	Avg score	**31.38**	33.66	32.73	33.86	51.44
5. ASR = 60%	Karate	**50.39**	51.33	51.70	52.42	63.70
	Krebs	**47.39**	48.49	47.52	48.64	62.99
	Airport	**50.73**	51.98	52.03	52.23	63.66
	Crime	**46.48**	49.33	49.08	48.91	61.13
	Power	**10.33**	13.35	10.58	13.61	34.34
	Oregon1	**38.50**	40.87	40.41	40.56	58.25
	Avg score	**40.64**	42.56	41.89	42.73	57.35
6. ASR = 50%	Karate	**59.90**	60.55	60.88	61.41	69.90
	Krebs	**57.61**	58.38	57.74	58.33	62.99
	Airport	**59.45**	60.32	60.39	60.52	63.66
	Crime	**57.59**	59.50	59.50	59.08	61.13
	Power	**16.54**	19.73	17.31	19.93	34.34
	Oregon1	**49.68**	51.58	51.28	51.33	58.25
	Avg score	**50.13**	51.69	51.18	51.77	57.35
7. ASR = 50% for the first 30% of nodes	Karate	**48.61**	50.38	49.76	50.57	50.79
	Krebs	**45.51**	47.16	45.78	47.38	53.00
	Airport	**48.78**	50.68	51.00	50.47	49.76
	Crime	**41.84**	48.17	46.90	47.12	50.06
	Power	**14.87**	17.79	16.32	17.86	27.04
	Oregon1	**41.26**	42.91	42.83	43.14	48.92
	Avg score	**40.15**	42.91	42.10	42.76	46.60
8. Random ASR	Karate	**35.12**	36.29	36.50	37.57	54.11
	Krebs	**30.39**	32.99	31.02	33.36	53.91
	Airport	**36.95**	38.58	38.67	38.99	55.24
	Crime	**27.90**	30.96	30.48	30.42	51.06
	Power	**5.17**	8.46	5.18	8.80	27.40
	Oregon1	**21.61**	24.23	23.52	23.86	48.79
	Avg score	**26.19**	28.56	27.55	28.79	48.42

**Table 5:** The computation time of different attack strategies (ms). Smaller values are better (best in bold).

Network	HBnnsAGP	FINDER	HBA	HDA
Karate	1.6	16.3	1.9	**0.5**
Krebs	3.6	36.6	4.6	**2.3**
Airport	82.3	218.3	211.0	**11.1**
Crime	552.1	369.3	4434.6	**49.1**
Power	6760.7	1397.9	78,119.9	**1796.8**
Oregon1	15,799.1	8641.5	477,802.8	**2065.9**

## Data Availability

Not applicable.
